# Disseminated tuberculosis among hospitalised HIV patients in South Africa: a common condition that can be rapidly diagnosed using urine-based assays

**DOI:** 10.1038/s41598-017-09895-7

**Published:** 2017-09-07

**Authors:** Andrew D. Kerkhoff, David A. Barr, Charlotte Schutz, Rosie Burton, Mark P. Nicol, Stephen D. Lawn, Graeme Meintjes

**Affiliations:** 1 Division of Infectious Disease, Department of Medicine, University of California San Francisco School of Medicine, San Francisco, CA USA; 2 Wellcome Trust Liverpool Glasgow Centre for Global Health Research, Institute of Infection and Global Health, University of Liverpool, Liverpool, UK; 3 Wellcome Trust Centre for Infectious Diseases Research in Africa (CIDRI-Africa), Institute of Infectious Disease and Molecular Medicine, University of Cape Town, Cape Town, South Africa; 4 Department of Medicine, Faculty of Health Sciences, University of Cape Town, Cape Town, South Africa; 5 Department of Medicine, Faculty of Medicine and Health Sciences, University of Stellenbosch, Cape Town, South Africa; 6 Southern African Medical Unit, Médecins Sans Frontières, Cape Town, South Africa; 7 Division of Medical Microbiology and Institute for Infectious Diseases and Molecular Medicine, Faculty of Health Sciences, University of Cape Town, Cape Town, South Africa; 8 The Desmond Tutu HIV Centre, Institute of Infectious Disease and Molecular Medicine, Faculty of Health Sciences, University of Cape Town, Cape Town, South Africa; 9 Department of Clinical Research, Faculty of Infectious and Tropical Diseases, London School of Hygiene and Tropical Medicine, London, UK

## Abstract

HIV-associated disseminated TB (tuberculosis) has been under-recognised and poorly characterised. Blood culture is the gold-standard diagnostic test, but is expensive, slow, and may under-diagnose TB dissemination. In a cohort of hospitalised HIV patients, we aimed to report the prevalence of TB-blood-culture positivity, performance of rapid diagnostics as diagnostic surrogates, and better characterise the clinical phenotype of disseminated TB. HIV-inpatients were systematically investigated using sputum, urine and blood testing. Overall, 132/410 (32.2%) patients had confirmed TB; 41/132 (31.1%) had a positive TB blood culture, of these 9/41 (22.0%) died within 90-days. In contrast to sputum diagnostics, urine Xpert and urine-lipoarabinomannan (LAM) combined identified 88% of TB blood-culture-positive patients, including 9/9 who died within 90-days. For confirmed-TB patients, half the variation in major clinical variables was captured on two principle components (PCs). Urine Xpert, urine LAM and TB-blood-culture positive patients clustered similarly on these axes, distinctly from patients with localised disease. Total number of positive tests from urine Xpert, urine LAM and MTB-blood-culture correlated with PCs (p < 0.001 for both). PC1&PC2 independently predicted 90-day mortality (ORs 2.6, 95%CI = 1.3–6.4; and 2.4, 95%CI = 1.3–4.5, respectively). Rather than being a non-specific diagnosis, disseminated TB is a distinct, life-threatening condition, which can be diagnosed using rapid urine-based tests, and warrants specific interventional trials.

## Introduction

Tuberculosis (TB) is a leading infectious cause of death worldwide, with an estimated 1.8 million deaths in 2015^1^; it also remains the leading cause of death in people living with HIV, contributing to 1 in 3 HIV-related deaths^2, 3^. HIV-associated TB is *disseminated* (involving multiple organ systems – typically spleen, liver, multiple lymph node groups, and/or kidneys) in 90% of post-mortem cases^4^. By contrast, HIV-seronegative adults dying with TB most commonly have sputum-smear positive disease^5, 6^ and age-associated comorbidities that put them at increased risk for death^7^. While extra-pulmonary pathology is seen in approximately 20% of HIV-uninfected adult TB patients in clinical studies, it tends to be anatomically compartmentalised, pauci-bacillary, and rarely fatal^8^. We therefore suggest that a clear distinction should be made between this (anatomically limited) extra-pulmonary TB disease and the disseminated disease seen in association with advanced HIV infection.

This HIV-associated disseminated TB disease phenotype implies ongoing blood stream distribution, which is supported by some clinical studies showing a high prevalence of *Mycobacterium tuberculosis* (MTB) blood culture positivity in HIV-associated TB^9, 10^. By contrast, MTB positive blood cultures are rarely seen in immune-competent patients, including those with extra-pulmonary disease (miliary TB accounts for <2% of TB cases in adult immune competent patients^11^), again emphasising the distinctiveness of HIV-associated disseminated TB.

Despite a presumably high bacillary burden, HIV- associated disseminated TB remains difficult to diagnose. HIV-associated TB often has a non-specific clinical presentation (i.e., without cough) and high proportions of sputum smear-negative and radiographically non-specific disease^12, 13^. Therefore, mycobacterial blood culture is considered the gold-standard diagnostic test for disseminated TB^14^. However, a lack of mycobacterial culture facilities in geographical areas with high HIV-TB burden, and the long incubation time before results are obtained, limits the practical use of MTB blood culture for diagnostic purposes^9, 15, 16^. Consequently, nearly half of the HIV-associated disseminated TB observed in post-mortem studies was undiagnosed prior to death^4^. Even when disseminated TB is diagnosed or treated empirically, there is substantial diagnostic delay and patients with such severe disease may not survive; the time from initiation of treatment to death is often only a few days, at which time culture conversion and mycobacterial suppression has not yet occurred, despite empiric therapy^17, 18^.

Because disseminated and/or MTB blood culture positive TB is difficult to diagnose ante-mortem, its clinical associations remain poorly described. Only recently has MTB bacteraemia been recognised as one of the most common causes of sepsis/severe sepsis in sub-Saharan Africa^9, 10^. Only a few studies have assessed relative mortality risk associated with a positive MTB blood culture^9, 19–21^. The relationship between MTB blood culture positivity and other markers of disseminated HIV-associated TB – such as lipoarabinomannan (LAM) antigenuria – remains unclear^22^. To date, no intervention trials have specifically focussed on MTB blood culture positive patients, and no international consensus guidelines address this condition.

In summary, HIV-associated disseminated TB is clinically distinct from anatomically limited extra-pulmonary TB seen in more immunocompetent adults. Disseminated TB is characterised by (and often used interchangeably with) MTB blood culture positivity. HIV-associated disseminated TB or MTB blood culture positive TB are under-recognised, which reflects diagnostic failures in HIV-infected patients. There is therefore a pressing need to better recognise and characterise HIV-associated disseminated TB or MTB blood culture positive TB to help facilitate interventional trials to reduce mortality.

We aimed to describe disseminated TB in a cohort of HIV-infected patients who underwent systematic investigation for TB disease within 24-hours of admission to hospital. Our objectives were the following: a) to ascertain the prevalence of MTB blood culture positivity, which could then be used as a gold-standard diagnostic for disseminated TB in this cohort; b) to determine clinical predictors of MTB blood culture positivity among HIV-associated TB inpatients; c) to assess if MTB blood culture positivity could be rapidly and reliably predicted by clinical or microbiological surrogates, including sputum and urine based diagnostics (i.e., calculate comparative diagnostic yield); d) to describe the clinical characteristics of HIV-associated TB inpatients in order to define a clinical phenotype for HIV-associated disseminated TB; e) to describe the associations between MTB blood culture, urine-based diagnostics, disseminated TB clinical phenotype and 90-day mortality.

## Methods

### Setting and patients

This prospective observational study is a sub-study of a previously reported parent study that investigated rapid urine-based approaches (urine LAM lateral-flow assay and urine Xpert) to diagnosing HIV-associated TB in inpatients^23^. The study was conducted from June 2012 to October 2013 at G.F. Jooste Hospital in Cape Town, South Africa, an approximately 200-bed district hospital serving several township communities in which there is a high prevalence of HIV and incidence of TB^24, 25^. All patients provided written informed consent in their first language. The study was approved by the human research ethics committees of the University of Cape Town and the London School of Hygiene & Tropical Medicine and was carried out in accordance with the Declaration of Helsinki.

Four days a week, consecutive adult patients (≥18 years old) who required medical admission were screened by a study nurse coordinator who ascertained all medical admissions in the previous 24-hour period from the ward register. Any patient previously testing HIV negative or who had an unknown/undocumented HIV status was offered rapid HIV testing and counselling^23^. Patients with previously or newly confirmed HIV-infection were approached for consent and recruited to the study regardless of symptomatology or reason for admission. This sub-study excluded those with a known TB diagnosis and patients receiving anti-TB therapy prior to current hospital admission.

### Procedures and samples

The nurse coordinator recorded all demographic and clinical details for recruited patients within 24 hours of admission. Specimen collection and TB investigations has been described previously^23, 26^. In brief, sputum, urine and blood specimens were obtained from all patients whenever possible within 24 hours of admission. Two sputum samples were requested from each patient – a spot specimen followed by an induced specimen. Urine samples were collected in single-use disposable bed-pans to avoid contamination (Litha Healthcare Group, Johannesburg). Venous blood was collected from all patients. The routine (non-study) medical team obtained additional samples (sputum and non-respiratory) during admission for TB investigations as clinically indicated.

### Laboratory procedures

All biochemistry and haematology tests (including full blood counts, CD4 cell counts, HIV viral loads and C-reactive protein [CRP] concentrations) were done at the Groote Schuur Hospital National Health Laboratory Service (NHLS). Microbiology specimens were also processed at the NHLS using standardised protocols. Sputum samples were tested using fluorescence microscopy, the Xpert MTB/RIF assay (Cepheid inc., Sunnyvale, CA, USA) and cultured in mycobacterial growth indicator tubes (MGIT; Becton Dickinson, Sparks, MD, USA). Urine was tested using Xpert on fresh urine samples (2.0 ml) as well as frozen samples (30–40 ml) that were thawed and concentrated via centrifugation^23, 27^. Frozen urine samples were also thawed and tested retrospectively using the Determine TB-LAM assay (Alere Inc., Waltham, MA, USA) according to manufacturer’s instructions; a grade 2 cut-off defined a positive result^28^. Venous blood samples were cultured using Myco F/Lytic culture (Becton Dickinson, Sparks, MD, USA). Culture isolates were identified as *M. tuberculosis* complex with the MTBDR*plus* line probe assay (Hain Lifescience, Nehren, Germany). All diagnostic tests were carried out by independent operators blinded to clinical information and other test results. TB results were made available to the routine clinical team to inform treatment decisions with the exception of urine LAM lateral-flow assay results, as this test was not endorsed for use by the South African Department of Health or the World Health Organization (WHO) at the time of the study.

### Clinical outcomes

Vital status at 90 days after study entry as well as subsequent hospital readmission within 90 days was determined using patient case notes and ward register data in addition to six electronic databases^29^. Those whose vital status could not be determined using one of the above sources were defined as lost-to-follow-up (LTFU).

### Data analysis

To assess the prevalence of disseminated TB, MTB blood culture positivity was considered the gold-standard test, and the analysis was restricted to:

1. patients with at least one mycobacterial blood culture result (n = 410), to determine prevalence amongst HIV-inpatients; and
2. patients with a new TB diagnosis during admission (n = 132, defined as detection of MTB from one or more clinical specimens from any anatomical site using culture and/or Xpert MTB/RIF), to determine prevalence amongst inpatients with HIV-associated TB.

To assess clinical predictors of MTB blood culture positivity, Wilcoxon rank-sum tests or Kruskal-Wallis tests were used to compare continuous variables and Chi-squared and Fisher’s exact tests to compare proportions. *A priori* variables and variables in the univariable analysis meeting a cut-off of p ≤ 0.1 were assessed in a multivariable logistic regression model. Anaemia severity was defined according to WHO criteria: none (haemoglobin concentration ≥13.0 g/dL for males, ≥12.0 g/dL for females), mild (11.0–12.9 g/dL for males, 11.0–11.9 g/dL for females), moderate 8.0–10.9 g/dL for males and females) or severe anaemia (<8.0 g/dL for males and females)^30^.

‘Diagnostic yield’ indicates the real-world performance of a given assay and is the proportion of all confirmed diagnoses that were made by a given test and therefore incorporates the ability to obtain specimens for that test in clinical practice as well as its sensitivity in the laboratory. To assess the ‘diagnostic yield’ the total number of patients with a positive MTB blood culture result was used as the denominator to calculate the comparative ‘diagnostic yield’ of TB diagnoses from different assays (microscopy, Xpert, LAM) on different samples types (sputum and urine)^26^. In addition, overall agreement between these tests (microscopy, Xpert, LAM), in which none is considered a gold-standard for disseminated TB, was assessed using pairwise Cohen’s Kappa statistics.

Principle components analysis (PCA) was used as a dimension reduction technique to summarise overall clinical variation amongst inpatients with HIV-associated TB on 10 major clinical variables (haemoglobin, mean corpuscular volume (MCV), red cell distribution width (RDW), serum sodium, C-reactive protein (CRP), albumin, HIV viral load, total lymphocyte count, CD4 cell count, and symptoms, selected *a priori*). Clinical phenotype, defined by the first two principle components (PC1, PC2) of this analysis, was then related to MTB diagnostic tests results. A composite score indexing number of tests positive from MTB blood culture, urine LAM and urine Xpert (giving a range of possible values between 0 and 3) was calculated for each patient and assessed for correlation with PC1 and PC2 by Spearman’s rank correlation coefficient. Association of PC1 and PC2 with 90-day mortality was assessed using logistic regression. For PCA, input clinical variables were selected *a priori*, missing data was imputed by Gibb’s sampling to avoid bias from data missing non-randomly, and varimax rotation was used^31^. All statistical tests were 2-sided at α = 0.05, and were carried out in RStudio version 0.99.892 or STATA version 12.0.

## Results

Of 609 HIV-positive adults admitted to the adult medical ward, 585 (96.1%) were enrolled in the study and systematically investigated for TB. Patients with a known TB diagnosis on admission and/or currently receiving TB treatment were excluded (n = 158). Of the 427 remaining patients, 17 did not have blood samples available for mycobacterial culture; thus 410 (96.0%) had MTB blood culture results available and were included. These patients were predominantly women (n = 249; 61%) with advanced immunodeficiency (median CD4 count, 150 cells/uL) and anaemia (median haemoglobin, 9.6 g/dL) (Table 1 **)**.

**Table 1:** Baseline characteristics stratified according to TB status and *M. tuberculosis* blood culture result

	All patients (n = 410)	TB-positive | positive BC (n = 41)	TB-positive | negative BC (n = 91)	No TB (n = 278)	P-value*	P-value**
**Age**	36.4 (28.9–42.4)	31.1 (26.3–38.2)	34.7 (28.0–40.8)	37.1 (30.0–44.0)	0.002	0.14
**Female**	249 (60.7)	26 (63.4)	58 (63.7)	165 (59.4)	0.71	0.97
**New HIV diagnosis on admission**	77 (18.8)	13 (31.7)	18 (19.8)	46 (16.6)	0.07	0.14
**ART status**						
ART-naïve	170 (41.5)	22 (53.7)	45 (49.5)	103 (37.1)	0.11	0.74
Current ART use	172 (42.0)	13 (31.7)	35 (38.5)	124 (44.6)		
ART-interrupted	68 (16.6)	6 (14.6)	11 (12.1)	51 (18.4)		
If currently on ART, treatment duration (years)^a^	1.6 (0.5–3.5)	0.7 (0.0–2.5)	1.4 (0.2–4.1)	1.6 (0.5–3.6)	0.25	0.29
**CD4 counts** (cells/uL)^b^						
Median (IQR) cells/µL	150 (55–312)	42 (29–127)	102 (41–225)	191 (74–388)	<0.001	0.003
<50	90 (22.1)	24 (58.5)	26 (28.9)	40 (14.4)	<0.001	0.001
50–99	62 (15.2)	3 (7.3)	18 (20.0)	41 (14.8)		
100–149	52 (12.8)	7 (17.1)	11 (12.2)	34 (12.3)		
150–199	41 (10.1	5 (12.2)	10 (11.1)	26 (9.4)		
≥200	163 (40.0)	2 (4.9)	25 (27.8)	136 (49.1)		
**HIV viral load** (log copies/mL)^c^						
Median (IQR)	4.4 (1.6–5.5)	5.3 (4.2–6.1)	4.8 (2.7–5.6)	4.0 (1.6–5.4)	<0.001	0.025
Virally suppressed (<400 copies/mL)	121 (30.3)	4 (10.8)	21 (23.9)	96 (35.0)	0.003	0.049
**Haemoglobin levels (g/dL)** ^d^						
Median (IQR)	9.6 (7.7–11.6)	7.7 (6.3–8.6)	8.9 (7.0–11.1)	10.2 (8.2–12.2)	<0.001	0.002
No/mild anaemia	138 (34.2)	2 (4.9)	25 (27.5)	111 (40.8)	<0.001	0.002
Moderate/severe anaemia	266 (65.8)	39 (95.1)	66 (72.5)	161 (59.2)		
**White cell count (**×10^9^ cells/L**)** ^**d**^	7.3 (4.9–10.4)	7.2 (5.0–10.1)	7.2 (4.7–9.4)	7.4 (5.1–11.0)	0.34	0.86
**Total lymphocyte count (**×10^9^ cells/L)^e^	1.02 (0.65–1.69)	0.64 (0.42–0.87)	0.94 (0.53–1.32)	1.13 (0.74–1.86)	<0.001	0.58
**Platelet count** (×10^9^ cells/L)^f^	260 (185–356)	207 (123–303)	261 (192–379)	263 (187–360)	0.031	0.012
**Albumin concentration** (g/L)^k^	20 (16–27)	18 (13–21)	20 (17–25)	23 (18–28)	0.011	0.06
**Sodium concentration** (mEq/L)^m^	133 (129–136)	130 (127–133)	130 (126–134)	134 (130–136)	<0.001	0.08
**Estimated GFR** (mL/min/1.73 m^2^)^b^	125 (18–227)	126 (80–167)	129 (95–166)	122 (86–157)	0.54	0.96
**C-reactive protein** (mg/L)^g^	72 (17–157)	138 (107–195)	99 (52–174)	53 (12–135)	<0.001	0.002
**Symptoms**						
Cough ≥2 weeks^b^	31 (7.6)	7 (17.1)	10 (11.1)	14 (5.1)	0.010	0.35
Current cough^b^	192 (47.1)	26 (63.4)	53 (58.9)	113 (40.8)	0.001	0.62
Haemoptysis^h^	29 (7.4)	19 (7.2)	5 (5.7)	5 (13.2)	0.33	0.15
Current fever^i^	60 (14.7)	8 (20.0)	15 (16.7)	37 (13.4)	0.46	0.54
Current night sweats^b^	164 (40.2)	23 (56.1)	44 (48.9)	97 (35.0)	0.006	0.44
Current reported weight loss^j^	179 (43.8)	25 (61.0)	45 (50.0)	109 (39.2)	0.013	0.24
Positive WHO symptom screen^b^	375 (91.7)	41 (100)	86 (95.6)	248 (89.2)	0.014	0.31
**TB characteristics**						
History of previous TB^a^	191 (46.7)	15 (36.6)	33 (36.7)	143 (51.4)	0.020	0.99
TB clinically suspected^#^	207 (53.5)	29 (72.5)	64 (75.3)	114 (43.5)	< 0.001	0.74

Data are median (IQR) or number (%). Percentages are column percentages. Abbreviations: ART = antiretroviral therapy, GFR = glomerular filtration rate, HIV = human immunodeficiency virus, IQR = interquartile range, TB = tuberculosis. a = 172 results available, b = 408 results available, c = 399 results available, d = 404 results available, e = 212 results available, f = 403 results available, g = 396 results available, h = 391 results available, i = 407 results available, j = 409 results available, k = 110 results available, m = 334 results available. *P-value comparison across three groups (n = 410). **P-value compares those with and without positive mycobacterial blood cultures among those with confirmed TB (n = 132). ^#^Hospital clinician suspected TB at time of admission. Estimated glomerular filtration rate was calculated with the Modification of Diet in Renal Disease (MDRD) Study equation 1⁄4 175 (creatinine) ^1.154^ (Age) ^0.203^ (0.742 if female) (1.212 if African).

### Prevalence of TB and MTB blood culture positivity

TB was newly diagnosed (detection of MTB from one or more clinical specimens from any anatomical site using culture and/or Xpert MTB/RIF) in 132/410 (prevalence, 32.2% [95%CI, 27.7–37.0]) HIV-infected in-patients. Overall, 41 patients had a positive MTB blood culture, which accounted for 10.0% (95%CI, 7.3–13.3) of all HIV-infected inpatients and nearly one-third (n = 41/132; 31.1%) of all HIV-infected inpatients with newly diagnosed TB (‘TB positive | blood culture positive’). Thus, 91 HIV-infected inpatients had new active TB disease and were mycobacterial blood culture negative (‘TB positive | blood culture negative’), and 278 had no microbiological evidence of TB disease (‘no TB’) (Table 1). No patients had non-tuberculous mycobacteria isolated from blood cultures.

### Predictors of MTB blood culture positivity

Patients with positive MTB blood cultures had more advanced immunosuppression compared to patients with negative MTB blood cultures (median CD4 count = 42 cells/μL; interquartile range [IQR] = 29–127 cells/μL versus median 102 cells/ μL, IQR = 41–225 cells/μL); however, 14/41 (34.2%) had a CD4 count greater than 100 cells/μL (Table 1). Among MTB blood culture positive patients, 13/41 (31.7%) were currently receiving ART, 8/41 (19.5%) had been receiving ART for ≥6 months and 4/41 (9.8%) were virally suppressed. Notably, 95% of MTB blood culture positive patients had either moderate or severe anaemia (median haemoglobin, 7.7 g/dL). They also had higher C-reactive protein (CRP) concentrations (median, 138 mg/L) and all had a positive WHO-symptom screen (Tables 1 and 2 **)**. In multivariable analysis, younger age, lower CD4 cell counts, lower haemoglobin concentrations and higher CRP concentrations were independently associated with positive MTB blood culture (Table 2 **)**. When multivariable analysis was restricted to those with confirmed TB (n = 132), only lower haemoglobin concentrations and lower platelet counts were associated with positive MTB blood culture (data not shown).

**Table 2:** Multinomial logistic regression of risk factors for *M. tuberculosis* blood culture positivity*.

	Unadjusted RR (95%CI)	p-value	Adjusted RR (95% CI)	p-value
**Age, for each year increase**	0.95 (0.91–0.98)	0.004	0.95 (0.90–1.00)	0.040
**Female**	1.13 (0.58–2.22)	0.71		
**New HIV status**	2.21 (1.09–4.50)	0.036	0.91 (0.36–2.27)	0.83
**ART status**				
Current use	1.0	0.24		
Naïve	1.81 (0.88–3.74)			
Defaulted	1.18 (0.43–3.25)			
**Previous history of TB treatment**				
No	1.0	0.17		
Yes	0.63 (0.09–0.20)			
**Current cough**				
No	1.0	0.027	1.0	0.43
Yes	2.10 (1.08–4.09)		1.41 (0.59–3.36)	
**Current weight loss**				
No	1.0	0.012	1.0	0.14
Yes	2.17 (1.12–4.20)		4.20 (0.70–25.39)	
**Current fever**				
No	1.0	0.38		
Yes	1.47 (0.64–3.36)			
**Current night sweats**				
No	1.0	0.030	1.0	0.25
Yes	2.05 (1.07–3.93)		0.33 (0.05–2.01)	
**CD4 (cells/μL), for every 50 unit decrease**	1.61 (1.29–2.01)	<0.001	1.32 (1.05–1.66)	0.004
**Viral load (copies/mL), for each log unit increase**	1.50 (1.18–1.90)	0.003	1.20 (0.89–1.63)	0.23
**Haemoglobin (g/dL), for each unit decrease**	1.40 (1.22–1.60)	<0.001	1.30 (1.09–1.56)	0.003
**Platelet (×10** ^**9**^ **cells/L), for each 10 unit decrease)**	1.03 (1.01–1.06)	<0.001	1.03 (0.99–1.06)	0.10
**Sodium (mmol/L), for each 1 unit decrease**	1.06 (0.99–1.14)	0.11		
**CRP (mg/L), for each 10 unit increase**	1.06 (1.03–1.10)	<0.001	1.06 (1.01–1.10)	0.013
**eGFR (mL/min/1.73** **m** ^**2**^ **), for each unit decrease**	0.99 (0.94–1.04)	0.60		

^*^Among 410 patients with mycobacterial blood culture results available.

### Outcomes in patients with positive MTB blood culture

Patients with positive MTB blood culture had poor outcomes compared to those with negative blood cultures (Table 3 **)**. Nearly 60% of those with positive MTB blood culture required hospital readmission within 90 days and approximately 44% received at least one blood transfusion within 3-months. In total 18/132 (13.6%) TB patients died within 90 days and 9/18 (50.0%) had a positive mycobacterial blood culture. Thus, 9/41 (22.0%) with positive MTB blood cultures died within 90 days and these patients had 2.2-times (95%CI, 1.0–5.2) increased risk of death within 90 days compared to HIV-associated TB patients with negative mycobacterial blood cultures. The median time to death in patients with a positive MTB blood culture was 20 days (range 6–88 days) from admission.

**Table 3:** Clinical outcomes at 90 days stratified according to TB status and *M. tuberculosis* blood culture result

	All patients (n = 410)	TB-positive | positive BC (n = 41)	TB-positive | negative BC (n = 91)	No TB (n = 278)	P-value
Required hospital readmission	123 (30.0)	24 (58.5)	21 (23.1)	78 (28.1)	<0.001
Required blood transfusion	52 (12.7)	18 (43.9)	7 (7.7)	27 (9.7)	<0.001
Died	48 (11.7)	9 (22.0)	9 (9.9)	30 (10.8)	0.096
Lost to follow-up	29 (7.1)	1 (2.4)	12 (13.2)	16 (5.8)	0.036
Died or Lost to follow-up	77 (18.8)	10 (24.4)	21 (23.1)	46 (16.6)	0.24

Number (%) are shown. P-value comparison across three groups (n = 410).

### Diagnostic yield of rapid TB diagnostic tests for detecting TB in those with positive MTB blood culture

The median time to mycobacterial blood culture positivity was 26 days (IQR, 23–31 days). Because of the delayed time to culture positivity, 5 of the 9 deaths occurred before the culture results were available - demonstrating the need for rapid TB diagnostics in those with a positive MTB blood culture.

**Figure 1:**
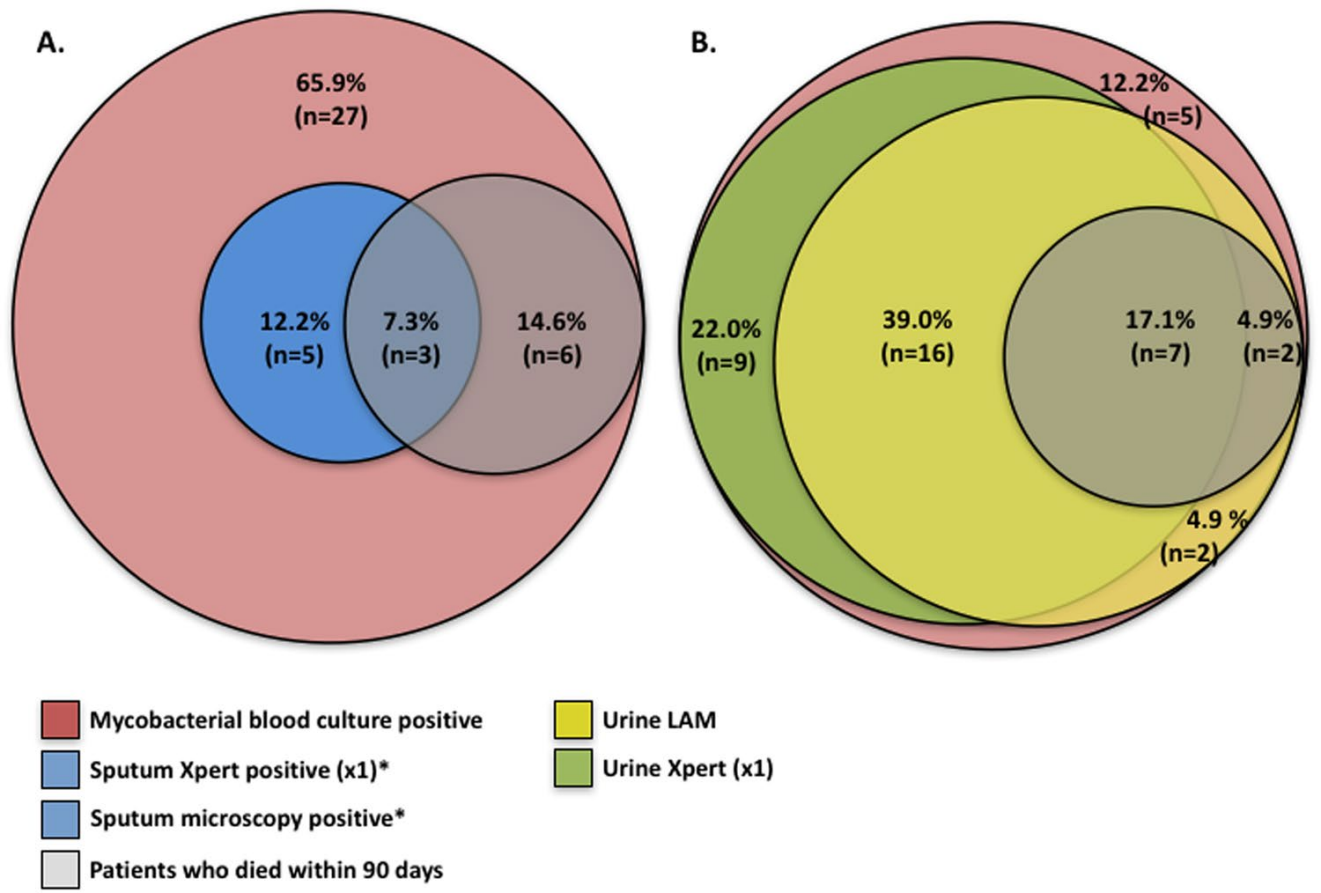
Venn diagram showing the proportions of *Mycobacterium tuberculosis* blood culture positive patients (n = 41) who had a TB diagnosis made by rapid microbiological tests. (**A**) Sputum-based diagnostics and (**B**). Urine-based diagnostics. Percentages represent the proportion of patients with positive mycobacterial blood culture diagnosed by a respective test and ‘n’ is the actual number of patients with positive mycobacterial blood culture diagnosed by a respective test. Any proportion (n) within the red portion was not diagnosed by any test, i.e. was exclusively detecting using mycobacterial blood culture. AFB = acid fast bacilli, LAM = Lipoarabinomannan. *Both sputum Xpert and sputum microscopy had identical diagnostic yield. Sputum microscopy and sputum Xpert had identical diagnostic yield for *Mycobacterium tuberculosis bacteraemia and both tests diagnosed n* = *3/9 Mycobacterium tuberculosis* blood culture positive *patients who died within 90 days*.

We calculated the comparative diagnostic yield of rapid microbiological assays for detecting TB in patients with positive MTB blood culture (Table 4). Rapid sputum-based diagnostics had limited utility for identifying patients with positive MTB blood culture, mainly due to the inability of very sick patients to provide sputa. Only 10/41 (24.4%) patients with positive MTB blood culture were able to produce a sputum sample within 24 hours of admission despite use of sputum induction. Thus, sputum smear microscopy and/or Xpert were only able to identify TB in 8/41 (19.5% (95%CI, 8.8–34.9)), including only 3 of 9 patients who died within 90 days **(**Table 4 and Fig. 1a **)**.

**Table 4:** Overview of diagnostic yield of rapid microbiological assays for tuberculosis stratified by mycobacterial blood culture result and vital status at 90 days (among n = 132 patients with newly diagnosed tuberculosis with mycobacterial blood culture results).

Assay	Diagnostic yield for newly diagnosed TB				Diagnostic yield for TB-related deaths			
	Blood culture positive (n = 41)		Blood culture negative (n = 91)		Blood culture positive (n = 41)		Blood culture negative (n = 91)	
	Number with new TB	Diagnostic yield for new TB *% (95%CI)*	Number with new TB	Diagnostic yield for new TB *% (95%CI)*	Number of patient dying within 90 days	Diagnostic yield in those dying within 90 days *% (95% CI)*	Number of patients dying within 90 days	Diagnostic yield in those dying within 90 days *% (95% CI)*
Overall	41	100	91	100	9	100	9	100
**Sputum**								
AFB smear	8	19.5 (8.8–34.9)	17	18.7 (11.3–28.2)	3	33.3 (7.5–70.1)	1	11.1 (0.2–48.2)
Xpert × 1	8	19.5 (8.8–34.9)	25	27.5 (18.6–37.8)	3	33.3 (7.5–70.1)	2	22.2 (2.8–60.0)
Xpert × 2	8	19.5 (8.8–34.9)	27	29.7 (20.5–40.2)	3	33.3 (7.5–70.1)	2	22.2 (2.8–60.0)
Either AFB smear or Xpert × 2	8	19.5 (8.8–34.9)	25	27.5 (18.6–37.8)	3	33.3 (7.5–70.1)	2	22.2 (2.8–60.0)
**Urine**								
LAM Grade 2	27	65.9 (49.4–79.9)	27	29.7 (20.5–40.2)	9	100 (66.4–100)	4	44.4 (13.7–78.8)
LAM Grade 1	32	78.0 (62.4–89.4)	30	33.0 (23.5–43.6)	9	100 (66.4–100)	6	66.7 (29.9–92.5)
Xpert non-concentrated	22	53.7 (37.4–69.3)	34	37.4 (27.4–48.1)	4	44.4 (13.7–78.8)	4	44.4 (13.7–78.8)
Xpert concentrated	32	78.0 (62.4–89.4)	45	49.5 (38.8–60.1)	7	77.8 (40.0–97.2)	8	88.9 (51.8–99.7)
Any urine Xpert	32	78.0 (62.4–89.4)	52	57.1 (46.3–67.5)	7	77.8 (40.0–97.2)	8	88.9 (51.8–99.7)
LAM Grade 2 and non-concentrated Xpert	32	78.0 (62.4–89.4)	43	47.3 (36.7–58.0)	9	100 (66.4–100)	6	66.7 (29.9–92.5)
LAM Grade 2 and concentrated Xpert	36	87.8 (73.8–95.9)	41	45.1 (34.6–55.8)	9	100 (66.4–100)	8	88.9 (51.8–99.7)
LAM grade 2 and any urine Xpert	36	87.8 (73.8–95.9)	55	60.4 (49.6–70.5)	9	100 (66.4–100)	8	88.9 (51.8–99.7)

Abbreviations: AFB = acid fast bacilli, LAM = Lipoarabinomannan.

Diagnostic yield is defined as the number of positive test results for given assay (numerator; by row) within the total set of patients (denominator; by column).

Rapid urine diagnostics performed favourably in patients with positive MTB blood cultures (Table 4 and Fig. 1b). The urine LAM lateral-flow assay identified 27/41 (65.9%, 95%CI = 49.4–79.9) of MTB blood culture positive patients, while Xpert testing of non-concentrated and concentrated urine samples identified MTB in 22/41 (53.7% 95%CI, 37.4–69.3) and 32/41 (78.0% 95%CI, 62.4–89.4), respectively. Urine LAM diagnosed TB in all 9 patients (100%) with positive MTB blood culture who subsequently died within 90 days of study entry, while Xpert testing on non-concentrated and concentrated urine samples identified MTB in 4/9 (44.4%) and 7/9 (77.8%) of such patients, respectively. A testing strategy combining urine Xpert (on concentrated sample) and the urine LAM lateral-flow assay would rapidly detect TB in 87.8% (95%CI, 73.8–95.9) of patients with positive MTB blood cultures (Table 4 and Fig. 1b **)**.

### Agreement between TB diagnostic assays

Having established urine LAM lateral-flow assay and urine Xpert as rapid surrogates for MTB blood culture in the 41 patients with positive MTB blood cultures, we next explored how these tests related to each other in the wider cohort of HIV positive inpatients with confirmed TB (n = 132).

Urine LAM had moderate agreement with mycobacterial blood cultures (Kappa = 0.52). Urine Xpert run on concentrated urine samples had similar agreement with mycobacterial blood cultures (Kappa = 0.48). Notably, agreement between concentrated urine Xpert and the urine LAM lateral-flow assay was also moderate (Kappa = 0.56). By comparison, agreement of mycobacterial blood culture with sputum smear microscopy and Xpert was limited (Kappa = −0.02 and 0.26, respectively).

We therefore hypothesised that urine Xpert and urine LAM, in addition to being surrogates of MTB blood culture, were also independent markers of disseminated TB (as opposed to compartmentalized or isolated renal TB). To investigate this we assessed how these test results related to clinical phenotypes within the cohort of patients with HIV-associated TB.

### HIV-associated TB clinical phenotypes

**Figure 2:**
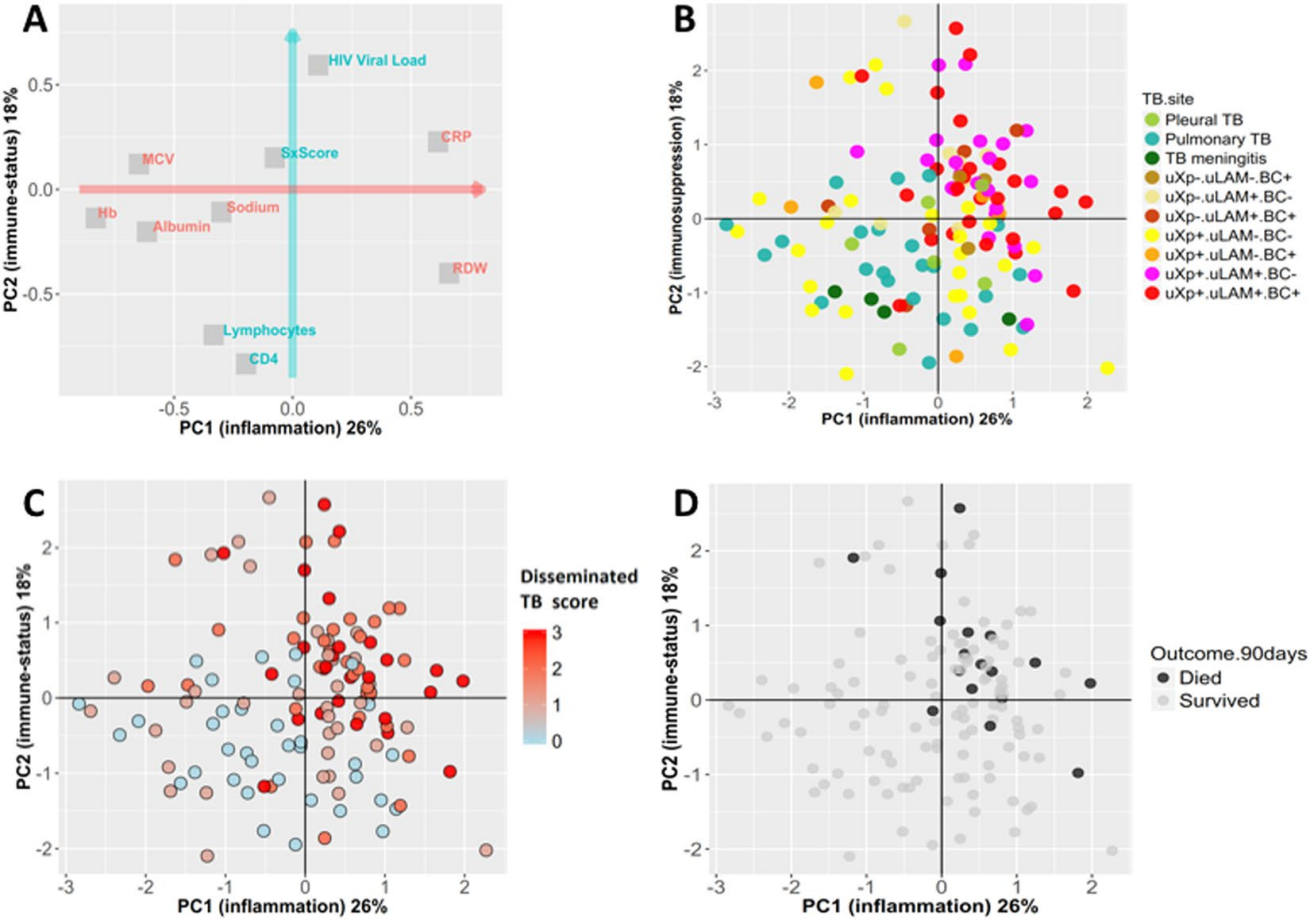
Principle components analysis (PCA) showing main dimensions of variation in laboratory and clinical variables for patients diagnosed with TB. The PCA was constructed with 10 major laboratory and clinical variables [haemoglobin, mean corpuscular volume (MCV), red cell distribution width (RDW), serum sodium, C-reactive protein (CRP), albumin, HIV viral load, total lymphocyte count, CD4 + cell count, and a symptom score based on number of ‘TB symptoms’ present (cough, haemoptysis, night sweats, fever, weight loss)]. Correlation of each of these variables with the first two principle components (shown as coordinates on panel A) demonstrated that the first principle component (PC1, red arrow) of clinical variation was dominated by markers of systemic inflammation (e.g. CRP, albumin, haemoglobin) while the second principle component (PC2, blue arrow) captured variation in HIV disease and immune status. These PCs were rotated using the varimax method and are therefore orthogonal (independent), and explained 26% and 18% of variation in the 10 clinical variable respectively. Although not used to construct these PCs, site of TB disease groupings clustered distinctively on these dimensions (panel B, each point is PC1 and PC2 coordinates of an individual patient, n = 129). PC1 and PC2 varied significantly overall by TB site (Kruskal-Wallis p = 0.003 and p < 0.001 respectively). Total number of positive tests out of the three assays {urine LAM, urine Xpert, M. tuberculosis blood culture} was associated with greater inflammation and immunosuppression (panel C). Compartmentalised extra-pulmonary TB in absence of dissemination (i.e. pleural TB or TB meningitis with negative urine LAM, urine Xpert and MTB blood culture) had significantly lower PC1 and PC2 values compared to disseminated TB. TB 90-day mortality was also strongly associated with “inflammation” and “immunosuppression” (panel D); a logistic regression model containing both PC1 and PC2 as independent variables showed adjusted odds ratios for mortality of 2.6 (95%CI 1.3–6.4, p = 0.017) and 2.4 (95%CI 1.3–4.5, p = 0.005) per one unit (one standard deviation) increase in PC1 and PC2 respectively. uXp = urine Xpert; uLAM = urine LAM; BC = MTB blood culture. Suffix “−” = test negative; suffix “ +” = test positive.

Principal components analysis (PCA) was used to map the different TB diagnostic results on to the main dimensions of clinical variation among 129 patients with confirmed TB and complete MTB blood culture, urine LAM lateral flow assay and urine Xpert results. The PCA was constructed with 10 a priori defined major clinical variables (Fig. 2A). The first principal component (PC1) summarised 26% of variation; in particular PC1 captured variation in markers of systemic inflammation, including CRP, haemoglobin and albumin levels. The second principal component (PC2) summarised a further 18% of clinical variation, and represented variation in markers of HIV disease and immune-status (HIV viral load, total lymphocyte and CD4 count). PC1 and PC2 were therefore treated as new, composite, independent variables representing “inflammation” and “immunosuppression” respectively. Consistent with logistic regression analysis, MTB blood culture positivity was strongly related to both the inflammation PC and immunosuppression PC, as was urine LAM lateral-flow assay positivity, while urine Xpert positive patients had a more variable phenotype (Fig. 2B).

Patients with pulmonary TB or anatomically limited forms of extra-pulmonary TB with negative mycobacterial blood culture or urine TB assays had similar PC1 and PC2 values (Fig. 2B; pleural TB [positive pleural fluid culture] or TB meningitis [positive cerebrospinal fluid culture] versus pulmonary TB, p = 0.67 and p = 0.53, respectively by Wilcoxon rank sum test). By contrast, urine Xpert, urine LAM lateral flow assay and MTB blood culture, were cumulative in their association with “immunosuppression” and “inflammation”; the total number of tests positive from urine Xpert, urine LAM and MTB blood culture (range 0–3) correlated with both “inflammation” (PC1) and “immunosuppression” (PC2) (p < 0.0001 for both; Fig. 2C).

**Figure 3:**
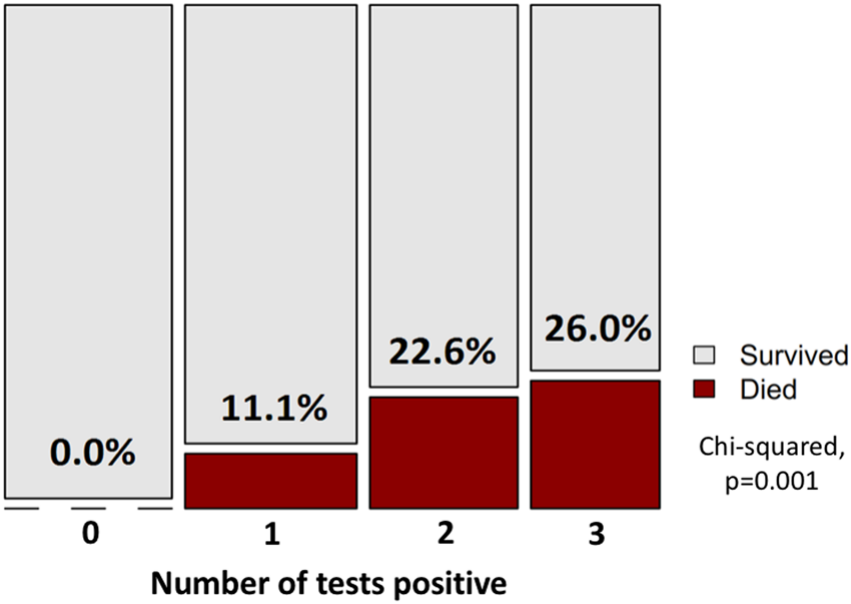
Plot of 90-day mortality outcome by number of TB tests positive (urine LAM, urine Xpert, and *M. tuberculosis* blood culture). *Note: area corresponds to absolute count of patients in category; proportion who died corresponds to height of red area.

Finally, the ‘inflammation’ and ‘immunosuppression’ composite variables derived from the PCA were independently associated with 90-day mortality (Fig. 2D). The total number of tests positive from urine Xpert, urine LAM and MTB blood culture was also strongly associated with higher proportional 90-day mortality, reaching 26.0% in those with a positive MTB blood culture, urine Xpert and urine LAM result (Fig. 3).

## Discussion

In this study, MTB blood cultures were positive in 10% of unselected HIV-infected South African adults requiring acute medical admission, and nearly one-third of those with newly diagnosed active TB disease. Patients with positive mycobacterial blood cultures had high rates of hospital re-admission and MTB bacteraemia was present in 22% of all deaths within 90 days of admission. This study adds to prior clinical studies from the last two decades demonstrating that disseminated TB – defined as culture positive MTB blood culture – is common amongst hospitalised HIV-infected patients in sub-Saharan Africa^9, 10, 32^.

The ability of the Determine TB-LAM lateral-flow assay, a 30-minute point-of-care test that costs less than $3 USD per result, to identify TB in the majority of patients with positive MTB blood cultures and identify all such patients who died within 90-days was notable. This study builds upon previous studies that have also demonstrated an association between urine LAM detection and MTB blood culture positivity^33, 34^. Our study also accords with the results of a recent randomized trial that demonstrated a mortality reduction associated with its use^35^ and strongly supports the recommendation by WHO that urine LAM should be used to aid the detection of HIV-associated TB among hospitalized patients with advanced immunodeficiency to allow for more rapid TB detection and initiation of potentially life-saving treatment^36^.

Furthermore, in this study, the addition of urine Xpert to urine LAM testing increased diagnostic yield for early identification of patients with positive MTB blood cultures, such that 88% of these patients could be identified using same-day diagnostic tests. In the near future, urine Xpert may have even greater sensitivity for disseminated TB through the more sensitive Xpert Ultra cartridge and may be more widely available at the point-of-care to rapidly inform treatment decisions via the Xpert Omni platform^37^.

HIV-associated disseminated TB is closely associated with, and possibly synonymous to, MTB blood culture positivity^14^ and MTB blood culture positivity remains the gold standard for diagnosis disseminated TB^14, 21^. Post-mortem studies have demonstrated that among the nearly 2 in 5 HIV patients that died who had evidence of TB, approximately 90% had disseminated, multi-organ disease and this was almost universally the primary cause of death^4^. This is substantially higher than the proportion of MTB blood culture positive cases among patients with HIV-associated TB who died in the present study (9/18 [50%] patients with confirmed TB who died within 90 days were blood culture positive at study entry). A blood culture only gives a snap-shot of bacteraemia at one time point, and bacteraemia in the absence of endo-vascular infection may be intermittent^38^; even when collected at the same time, multiple MTB blood cultures may increase yield^39, 40^. Consequently, a single blood culture, while highly specific, may have imperfect sensitivity for disseminated TB, underestimating the presence and extent of bacillary burden.

We found that urine LAM lateral-flow assay, urine Xpert and *M. tuberculosis* blood culture were cumulative measures of TB disease severity, correlating with systemic inflammation, immune-suppression, and death. We therefore speculate that these tests can report a measure of overall mycobacterial dissemination burden in patients with HIV-associated TB. MTB bacillary burden has been suspected to be a major driver defining the clinical spectrum and outcomes of TB disease, but has, to date, been difficult to measure^41, 42^. Blood and urine samples are accessible in all patients irrespective of clinically detectable extra-pulmonary pathology and can thus give an unbiased assessment of disease extent. A simple score based on these tests has potential utility as a quantitative marker of MTB bacillary burden for clinical scientists studying HIV-associated TB disease, if validated in future work.

Strengths of this study include enrolment of unselected, consecutively recruited HIV-infected adults requiring acute medical admission for any reason, which should make results generalizable to other hospitals in Southern and Eastern Africa where the burden of HIV-associated TB is high. Additionally, very thorough, systematic testing of patients for TB provided a reliable denominator against which the proportion of patients with HIV-associated TB with MTB blood culture positivity could be calculated^23^. Furthermore, mycobacterial blood culture results were available for >96% of patients. Our study did however, have limitations. Vital signs and blood lactate levels were not systematically recorded and thus we were unable to determine the proportion of patients with sepsis syndrome and septic shock due to MTB bacteraemia. Additionally, routine blood cultures were not systematically undertaken and thus we do not know what proportion MTB blood stream infection comprised of all blood stream infections in this cohort. Finally, TB treatment data was unavailable and we were therefore unable to investigate which patients with positive MTB blood cultures died despite early empirical therapy.

In conclusion, MTB blood culture positivity was identified in one out of every 10 HIV-infected patients requiring acute medical admission and was associated with half of HIV-associated TB deaths occurring within 90 days. Testing of urine samples obtained within 24 hours of admission using Xpert and the urine LAM lateral-flow assay identified TB in a large majority of those with positive blood cultures including all such patients who died within 90 days. MTB blood culture, urine Xpert and urine LAM results were strongly associated with major axes of clinical variation and mortality risk, and we postulate that these three tests in combination are a potential unbiased quantitative marker of MTB bacillary load in patients with HIV-associated disseminated TB. This study adds support to the roll out of urine-based point-of-care tests, especially the urine LAM lateral-flow assay, to rapidly identify those patients with disseminated disease as a mortality reduction strategy.
